# Enlarging Inflammatory Granulation Tissue After Endoscopic Submucosal Dissection at the Esophageal Inlet During Intensive Stricture Prophylaxis: A Case Report

**DOI:** 10.1002/deo2.70328

**Published:** 2026-04-13

**Authors:** Chihiro Tsurita, Naoya Tada, Akira Dobashi, Yukio Nishiya, Kosuke Sasuga, Mamoru Ito, Masato Nagaoka, Masayuki Shimoda, Kazuki Sumiyama

**Affiliations:** ^1^ Department of Endoscopy The Jikei University School of Medicine Tokyo Japan; ^2^ Department of Internal Medicine Division of Gastroenterology and Hepatology The Jikei University School of Medicine Tokyo Japan; ^3^ Department of Otolaryngology The Jikei University School of Medicine Tokyo Japan; ^4^ Department of Pathology The Jikei University School of Medicine Tokyo Japan

**Keywords:** endoscopic submucosal dissection, esophagus, granulation tissue, laryngoscope, stricture prophylaxis

## Abstract

Surveillance endoscopy in a 47‐year‐old woman with previous hypopharyngeal chemoradiotherapy and esophageal endoscopic submucosal dissection (ESD) revealed a 12‐mm superficial squamous cell carcinoma at the cervical esophagus near the inlet. ESD achieved en bloc resection with a half‐circumferential mucosal defect, and histopathology showed pT1a lamina propria mucosae with negative margins. For stricture prophylaxis, triamcinolone was injected into the post‐ESD defect, oral prednisolone was tapered over several months, and prophylactic endoscopic balloon dilation was performed three times at 3–4‐week intervals. Three months after ESD, a 10‐mm white‐coated lesion appeared at the inlet and progressively enlarged. Under intravenous sedation, detailed endoscopic assessments and biopsies were limited by the complex location, and the biopsy suggested neoplastic cells with marked degeneration. Due to progressive odynophagia and poor oral intake, endoscopic re‐evaluation under general anesthesia was performed. Using Sato's curved laryngoscope, the lesion was clearly visualized as a semipedunculated mass arising from the post‐ESD site and was completely resected for diagnosis and symptom relief. Histopathology revealed inflammatory granulation tissue without malignancy. Symptoms resolved promptly with no recurrence during 6 months of follow‐up. This case highlights the development of exuberant granulation tissue at the post‐ESD site in the cervical esophagus during intensive stricture prophylaxis. Although this rare finding likely reflects multiple overlapping factors, endoscopic evaluation and treatment were successfully performed while avoiding overtreatment.

## 1 Introduction

Endoscopic submucosal dissection (ESD) is an established minimally invasive treatment for superficial esophageal cancer that enables en bloc resection and precise histopathological assessments. However, post‐ESD management can be challenging when the lesion is located in the cervical esophagus near the inlet, where the lumen is narrow, and the anatomy is complex [1]. For extensive circumferential resections, stricture prevention may require local or oral steroids and/or endoscopic balloon dilation (EBD). When a new tumorous lesion develops at a post‐ESD site in this region, especially in patients with hypopharyngeal cancer and radiotherapy history, local recurrence must be considered, yet endoscopic and histopathological findings may become atypical. [2, 3] Herein, we report a rare enlarging tumorous lesion at the inlet that developed at a post‐ESD site that was successfully managed by endoscopic resection. We also discuss the potential contributing factors and technical considerations.

## 2 Case Report

A 47‐year‐old woman had undergone chemoradiotherapy (CRT) for hypopharyngeal cancer arising from the right pyriform sinus 4 years earlier, and ESD for thoracic esophageal cancer 3 years earlier. Surveillance endoscopy revealed a 12‐mm squamous cell carcinoma (SCC) in the cervical esophagus near the inlet, and the lesion was diagnosed as a superficial SCC. ESD was performed under general anesthesia because of the strong pharyngeal reflex and poor scope maneuverability; en bloc resection was achieved (Figure S1). Because the resected specimen measured 25 × 18 mm and the mucosal defect involved more than half of the circumference, a local injection of triamcinolone acetonide (40 mg) was administered. Oral prednisolone was initiated at 30 mg/day and gradually tapered by 5 mg every 2 weeks. Histopathology confirmed SCC limited to the lamina propria mucosae with negative margins. The muscularis mucosae was identified in the resected specimen, supporting an esophageal origin. To prevent postoperative strictures, prophylactic EBD was performed every 3–4 weeks for three sessions (Figure S2).

**FIGURE 1:**
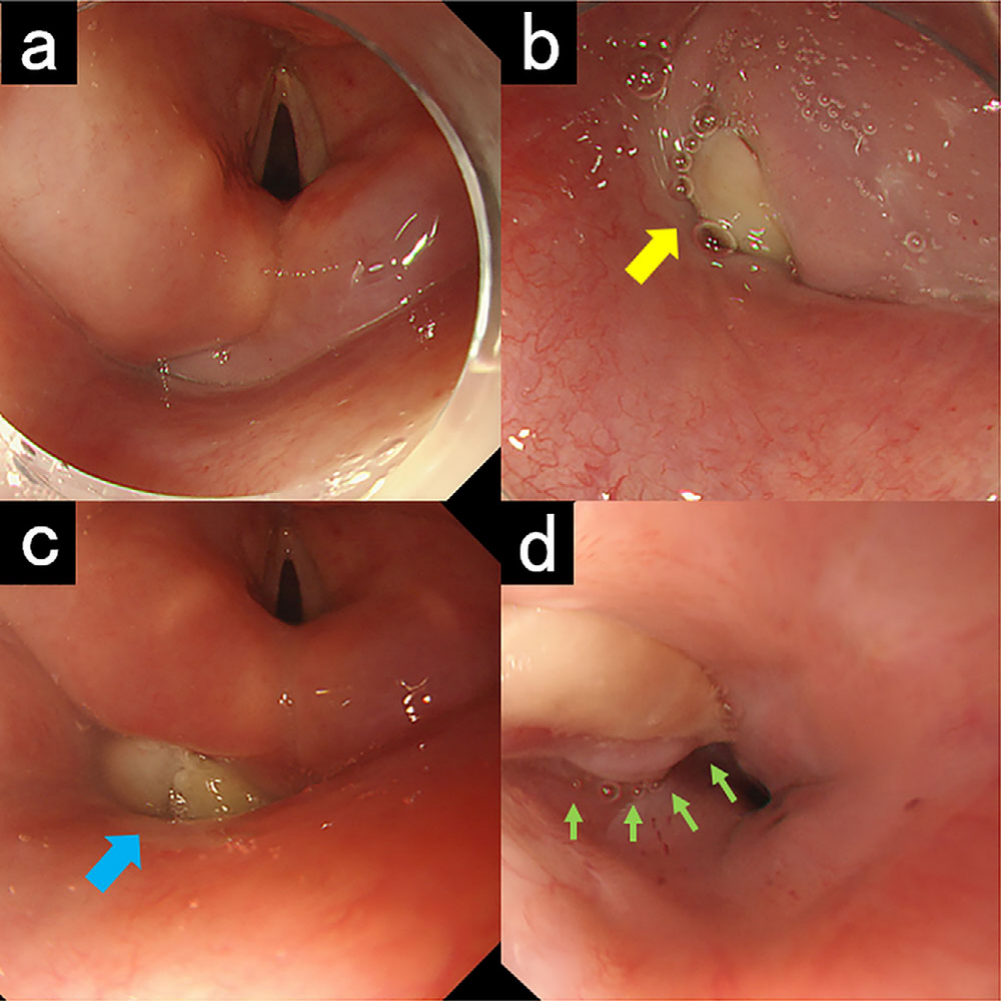
Three months after endoscopic submucosal dissection (a, b). (a) No obvious tumorous lesions were observed in the hypopharynx. (b) A white tumorous lesion was observed in the hypopharynx near the esophageal inlet (yellow arrow). Four months after endoscopic submucosal dissection (c, d). (c) A tumorous lesion was observed in the left pyriform sinus of the hypopharynx, which appeared slightly enlarged compared to the previous examination (blue arrow). (d) The tumorous lesion extended into the cervical esophagus (green arrows).

An esophagogastroduodenoscopy (EGD) conducted 3 months after ESD demonstrated an elevated lesion measuring approximately 10 mm with a white coating extending from the hypopharynx to the cervical esophagus; it was considered granulation tissue (Figures 1a, b). In contrast, a biopsy obtained by using a flexible laryngoscope suggested neoplastic cells with degenerative changes (Figure S3). A month later, EGD showed enlargement of the lesion, and repeated biopsies were performed (Figures 1c,d). Histopathology revealed a small number of AE1/AE3‐positive cells in the superficial layer. However, no definitive malignant atypia was observed (Figure S4). Although residual SCC could not be completely excluded, a definitive diagnosis was difficult because of the marked degenerative changes. Contrast‐enhanced computed tomography (CT) revealed an enhancing 20‐mm mass extending from the hypopharynx to the cervical esophagus (Figure S5). Due to progressive odynophagia and reduced oral intake, endoscopic re‐evaluation with biopsy under general anesthesia was planned to rule out malignancy, which could have required total laryngectomy.

**FIGURE 2:**
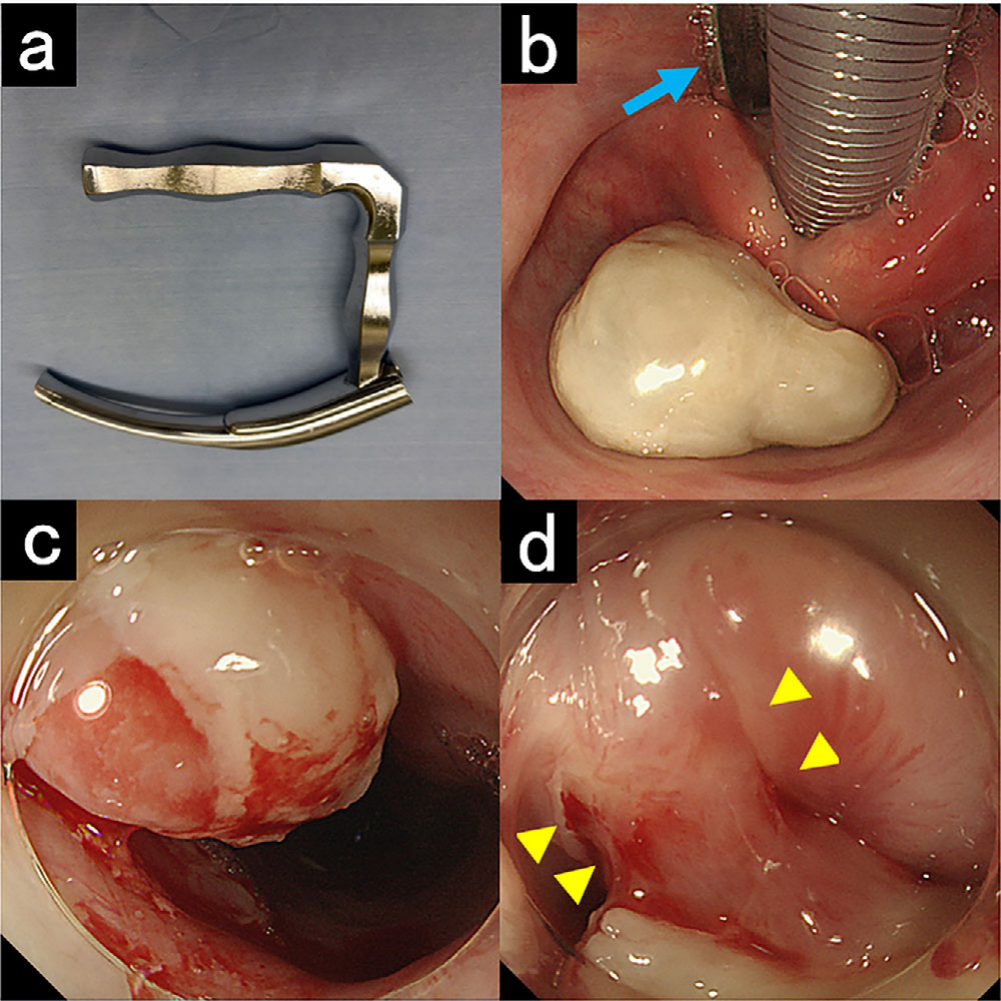
Endoscopic images obtained using Sato's curved laryngoscope under general anesthesia, exposing the larynx and esophageal inlet and allowing clear visualization of the lesion. (a) Sato's curved laryngoscope. (b) White mass protruding into the hypopharynx. Sato's curved laryngoscope (blue arrow) allows for clear visualization of the lesion. (c) Lesions at the esophageal inlet. (d) The base of the mass is semipedunculated (yellow arrowheads).

**FIGURE 3:**
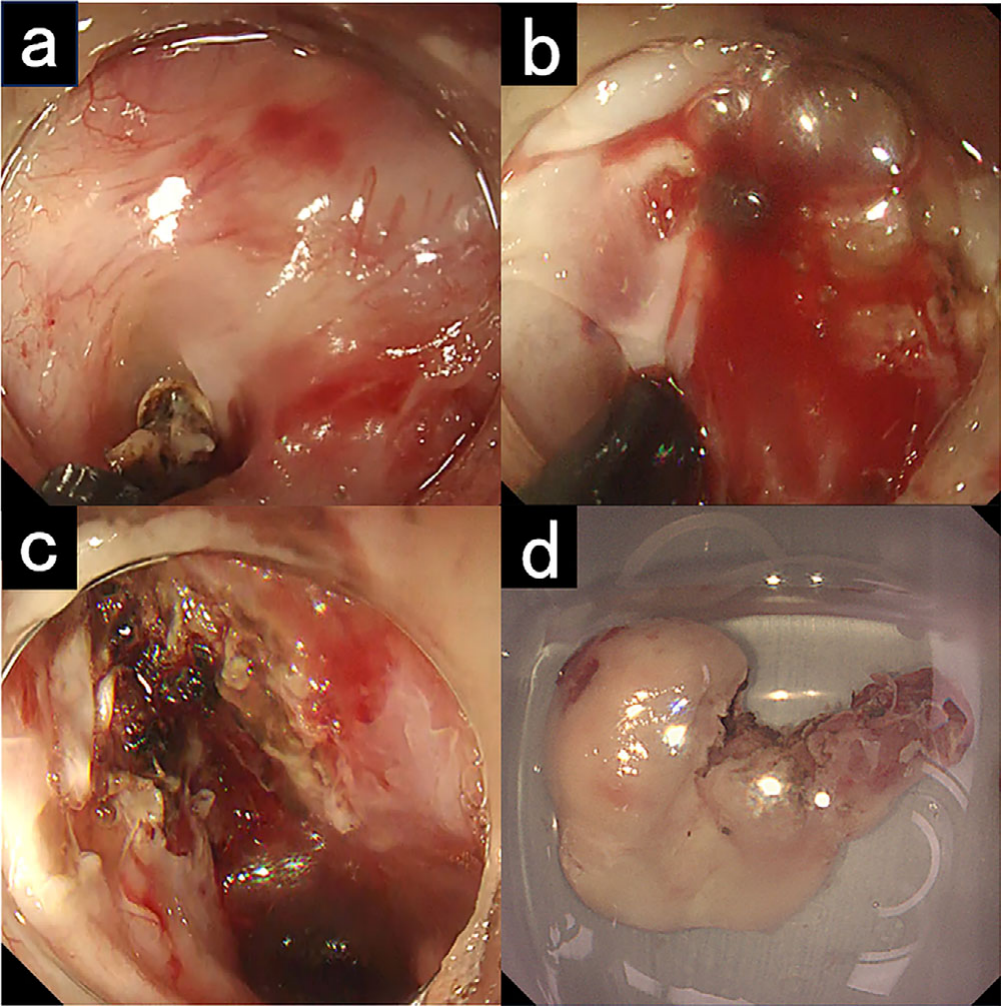
Endoscopic resection using an ITknife2. (a) Resection of the semipedunculated base of the lesion. (b) The lesion was highly vascular, resulting in minor bleeding during resection. (c) Post‐resection mucosal defect. (d) Resected specimen.

A Sato's curved laryngoscope (Nagashima Medical Instruments Co., Ltd., Tokyo, Japan) was used to expose the larynx and esophageal inlet, allowing for clear visualization of the lesion (Figure 2). The lesion appeared as a semipedunculated mass arising from a post‐ESD scar. The base was smooth, and no epithelial irregularities or abnormal vascular patterns were observed, suggesting a benign lesion. Endoscopic resection as a total biopsy was performed to obtain a definitive diagnosis and relieve symptoms. The base of the lesion was resected using an ITknife2 (Olympus Corporation, Tokyo, Japan) without submucosal injection. While frequent bleeding occurred because the lesion was highly vascular, complete resection was achieved within 5 min (Figure 3 and Video S1).

**FIGURE 4:**
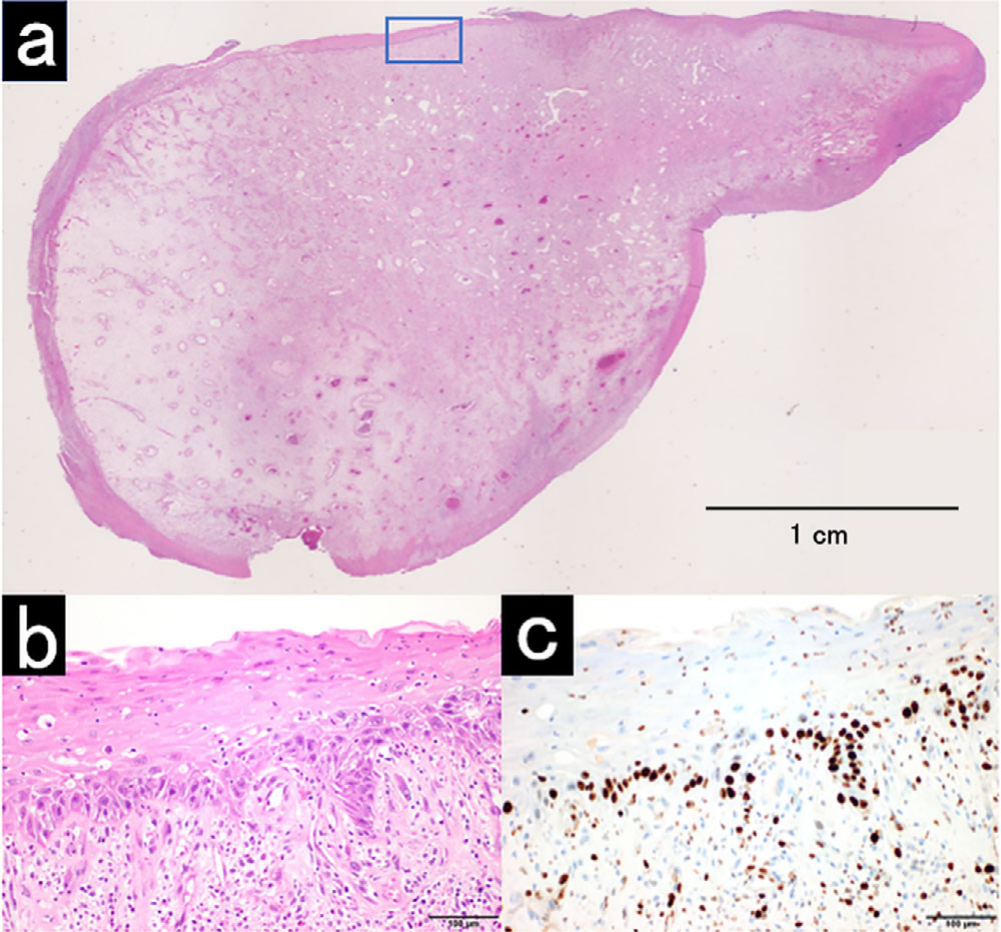
Inflammatory granulation tissue with atypical squamous epithelium and stromal cell proliferation. (a) Low‐power view of the resected specimen (hematoxylin and eosin stain). (b) Mild epithelial atypia with slight nuclear enlargement was observed from the basal layer to the second and third layers (×200). (c) MIB‐1–positive cells were confined to the basal and parabasal layers (×200).

Histopathology revealed inflammatory granulation tissue with fibroblast proliferation, neovascularization, and inflammatory cell infiltration, without evidence of malignancy. Mild epithelial atypia with slight nuclear enlargement was observed in the basal to parabasal layers (Figure 4). The stromal core showed diffuse positivity for vimentin, arguing against an epithelial neoplasm. MIB‐1–positive cells were confined to the basal and parabasal layers, consistent with reactive changes likely related to prior radiotherapy (Figure S6). The patient's pharyngeal pain disappeared immediately after resection. Oral intake was resumed the next day, and she was discharged on postoperative day 7 without complications. During 6 months follow‐up period, no recurrence or new lesions were observed.

## 3 Discussion

We present a rare instance of a large inflammatory granulation tissue developing at the esophageal inlet after ESD for cervical esophageal SCC. Although reports have described mass‐forming lesions arising from post‐ESD scars in the stomach and colorectum [4, 5], such lesions are uncommon after esophageal ESD. Such inflammatory granulation after esophageal ESD may pose clinical challenges in differentiating recurrence from a benign lesion and selecting therapeutic approaches for symptom relief.

During wound‐healing after esophageal ESD in the porcine model, inflammatory granulation tissue forms early after ulcer bed exposure and serves as a scaffold; proliferation and horizontal alignment of α‐smooth muscle actin–positive myofibroblasts then occur, followed by re‐epithelialization [6]. This granulation phase gradually resolves with healing; however, the disruption of this process may prolong granulation and result in exuberant tissue formation. Several factors may have influenced the formation of large granulation tissue at the post‐ESD site in the esophagus. First, the patient had previously undergone radiotherapy for hypopharyngeal cancer. Prophylactic irradiation of 40 Gy was delivered to the cervical lymph node regions, followed by a boost to the hypopharyngeal lesion to a total dose of 70 Gy. According to the treatment planning CT, the radiation field extended from the C1 to the Th3 vertebral levels. The cervical esophageal lesion in the present case was at the C5–C6 level and was therefore entirely within the prior radiation field. Such irradiation can cause long‐term microvascular damage, tissue hypoxia, and progressive fibrosis, resulting in impaired mucosal regeneration and abnormal wound‐healing responses [7, 8]. These radiation‐induced changes can persist for years and may render irradiated tissues prone to impaired wound‐healing and exaggerated reparative responses when subjected to additional insults, including endoscopic procedures. Second, although local and systemic steroid therapy is effective in preventing post‐ESD strictures, it may further delay wound‐healing by suppressing fibroblast proliferation, collagen deposition, and angiogenesis; interfering with granulation tissue maturation [9]. Local triamcinolone injection combined with oral steroid therapy may have contributed to the prolonged inflammation and exuberant granulation tissue formation. Third, repeated EBD performed for stricture prophylaxis before re‐epithelialization likely served as a source of ongoing mechanical trauma. Additionally, because the lesion was located in the cervical esophagus, swallowing‐related stimulation during meals may have been an additional irritant. Ongoing mechanical trauma before complete re‐epithelialization may prolong the proliferative phase of healing, which is characterized by fibroblast activity and angiogenesis, resulting in polypoid, mass‐like granulation tissue on an ESD scar [4, 9, 10]. The synergistic effects of radiation‐associated tissue fragility, steroid‐related impairment of wound‐healing, and repeated mechanical stimulation likely played a central role in the development of large granulation tissue.

Lesions at the esophageal inlet are often difficult to evaluate using flexible endoscopy owing to anatomical constraints. Moreover, general anesthesia may be required due to a strong pharyngeal reflex and limited maneuverability. Sato's curved laryngoscope enables stable exposure from the hypopharynx to the esophageal inlet, facilitating precise visualization and safe endoscopic resection [1]. In previously irradiated lesions, the histopathological interpretation of forceps biopsy can be challenging because radiotherapy can induce epithelial necrosis/atrophy and delayed reactive cellular atypia, potentially confounding assessment for recurrence [2, 3]. Herein, preoperative biopsy suggested a neoplastic lesion, but the findings were inconclusive. Total laryngectomy was considered a treatment option in the event of malignancy. Ultimately, however, minimally invasive endoscopic resection led to a definitive diagnosis and symptom relief.

The inflammatory granulation tissue after esophageal ESD may enlarge and mimic local recurrence, particularly in patients with a history of radiotherapy around the lesion and intensive stricture prophylaxis involving steroid therapy and repeated dilation. Awareness of this benign lesion is essential to avoid overtreatment when a new mass develops on a post‐ESD esophageal scar.

## Author Contributions

**Chihiro Tsurita**: writing of the original draft and literature research. **Naoya Tada**: endoscopic procedures, writing of the review and editing, and literature research. **Akira Dobashi**: clinical management (outpatient care), treatment planning, and writing – review and editing. **Yukio Nishiya**: clinical management (outpatient and inpatient care), treatment planning, and supporting endoscopic procedures. **Kosuke Sasuga**: pathological Assessment. **Mamoru Ito**: writing, review, and editing; **Masato Nagaoka**: treatment planning and overall clinical supervision. **Masayuki Shimoda**: pathological assessment, writing, review, and editing. **Kazuki Sumiyama**: endoscopic procedures, writing, review, and editing, and overall supervision.

## Funding

The authors have nothing to report.

## Ethics Statement

Not applicable.

## Conflicts of Interest

Kazuki Sumiyama is the deputy editor‐in‐chief of DEN Open. The remaining authors declare no conflicts of interest.

## Supporting information




**Figure S1**: (a) Narrow‐band imaging showing a superficial esophageal squamous cell carcinoma (SCC) at the esophageal inlet, appearing as a brownish area at the 12 o'clock position (yellow arrows).
(b) Lugol chromoendoscopy showing the lesion as an unstained (Lugol‐voiding) area at the 11 o'clock position (blue arrows).
(c) Mucosal defect after endoscopic submucosal dissection (ESD) involving more than half of the esophageal circumference (green arrows).
(d) Gross view of the formalin‐fixed resected specimen. The muscularis mucosae was identified throughout the specimen (red line indicates the SCC component).

**Figure S2**: (a) Post–endoscopic submucosal dissection ulcer involving more than half of the esophageal circumference on postoperative day 49. No obvious tumorous lesions were observed in post‐ESD mucosal defects.
(b) Endoscopic balloon dilation was performed.

**Figure S3**: Biopsy specimens showing degenerated tumor cells with irregularly enlarged nuclei and increased chromatin on hematoxylin and eosin staining (a, ×40; b, ×200). Figure (b) shows a higher‐magnification view of the blue boxed area in Figure (a).

**Figure S4**: Four months after ESD, repeat biopsy specimens showed a small number of AE1/AE3‐positive cells in the superficial layer. However, no AE1/AE3‐positive findings corresponding to definitive atypical cells were identified. Although residual SCC with treatment‐related effects could not be excluded, marked degeneration made it difficult to determine whether the lesion was benign or malignant. (a, Hematoxylin and eosin staining, ×40; b, Hematoxylin and eosin staining, ×200; c, AE1/AE3 immunostaining, ×200). Figures (b) and (c) show higher‐magnification views of the boxed area in Figure (a).

**Figure S5**: Contrast‐enhanced computed tomography images.
(c) Axial view showing an approximately 10‐mm enhancing mass (yellow circle).
(d) Sagittal view showing an approximately 20‐mm enhancing mass (red circle).

**Figure S6**: Immunohistochemical findings of the stromal core of the inflammatory granulation tissue with atypical squamous epithelium and stromal cell proliferation.
(a) Vimentin was diffusely positive (×200).
(b) Numerous MIB1‐positive cells were observed (×200).

**Video S1**: Endoscopic resection of a large semipedunculated lesion at the esophageal inlet.
