# Supplementary figures and images for: Enlarging Inflammatory Granulation Tissue After Endoscopic Submucosal Dissection at the Esophageal Inlet During Intensive Stricture Prophylaxis: A Case Report
Source: DEN Open. 2026 Apr 13;6(1):e70328. doi: 10.1002/deo2.70328 (PMC13071858; doi:10.1002/deo2.70328)

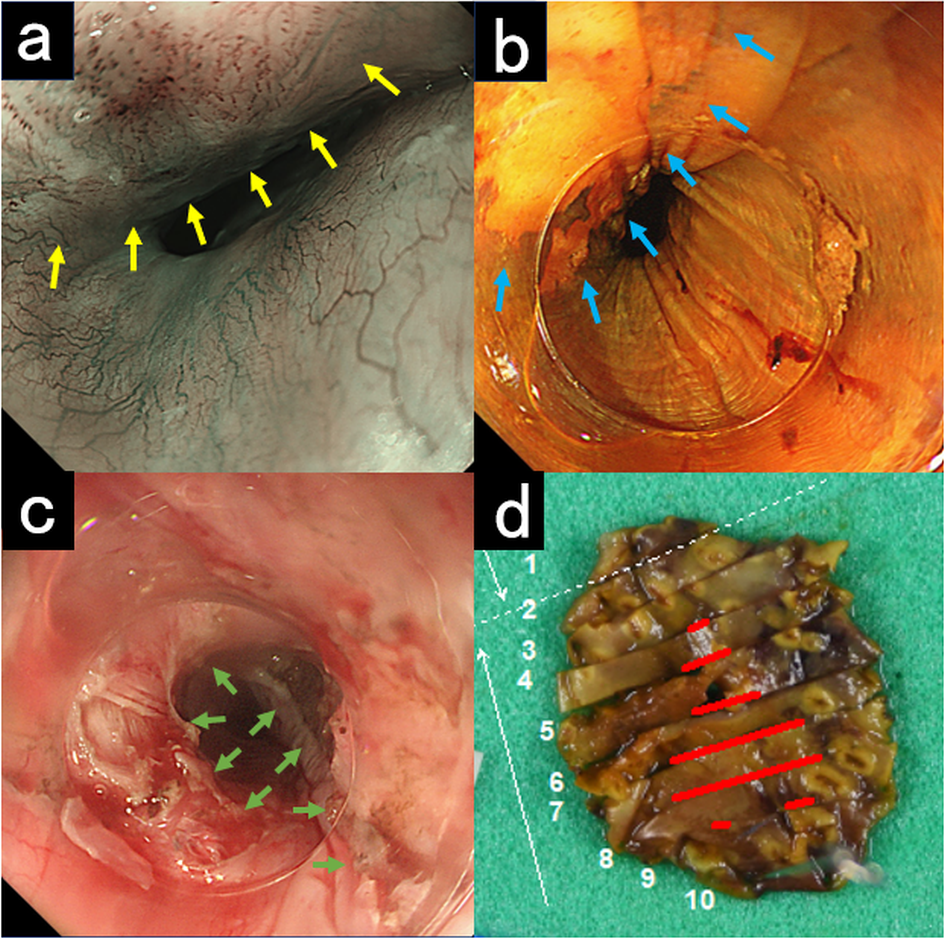

Supplement: Supplementary file 1 — Figure S1: (a) Narrow‐band imaging showing a superficial esophageal squamous cell carcinoma (SCC) at the esophageal inlet, appearing as a brownish area at the 12 o'clock position (yellow arrows). (b) Lugol chromoendoscopy showing the lesion as an unstained (Lugol‐voiding) area at the 11 o'clock position (blue arrows). (c) Mucosal defect after endoscopic submucosal dissection (ESD) involving more than half of the esophageal circumference (green arrows). (d) Gross view of the formalin‐fixed resected specimen. The muscularis mucosae was identified throughout the specimen (red line indicates the SCC component). [file DEO2-6-e70328-s004.tif]

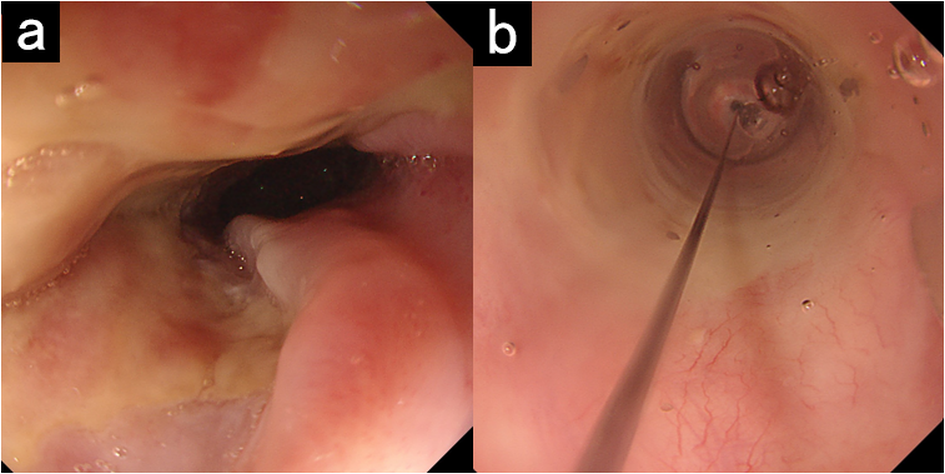

Supplement: Supplementary file 2 — Figure S2: (a) Post–endoscopic submucosal dissection ulcer involving more than half of the esophageal circumference on postoperative day 49. No obvious tumorous lesions were observed in post‐ESD mucosal defects. (b) Endoscopic balloon dilation was performed. [file DEO2-6-e70328-s003.tif]

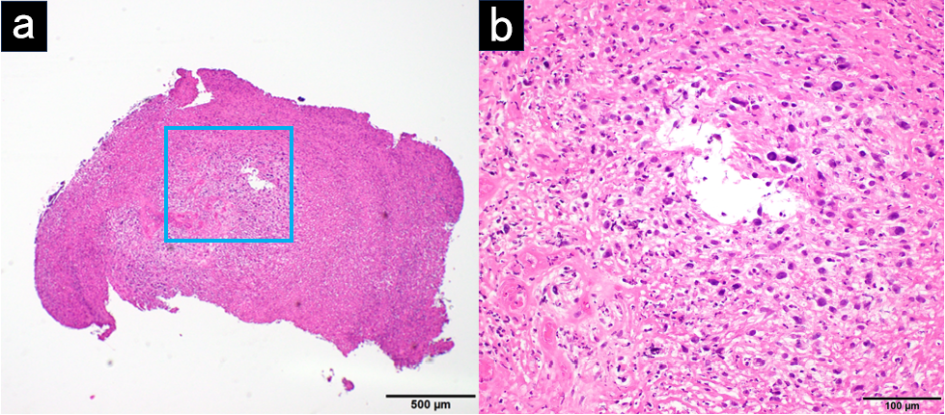

Supplement: Supplementary file 3 — Figure S3: Biopsy specimens showing degenerated tumor cells with irregularly enlarged nuclei and increased chromatin on hematoxylin and eosin staining (a, ×40; b, ×200). Figure (b) shows a higher‐magnification view of the blue boxed area in Figure (a). [file DEO2-6-e70328-s006.tif]

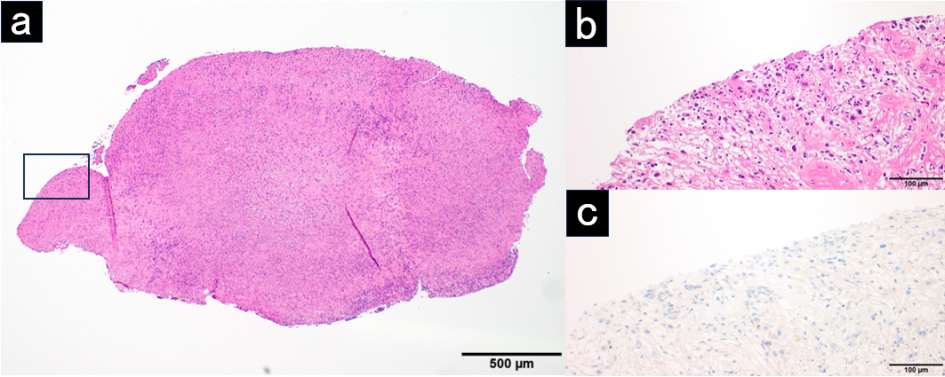

Supplement: Supplementary file 4 — Figure S4: Four months after ESD, repeat biopsy specimens showed a small number of AE1/AE3‐positive cells in the superficial layer. However, no AE1/AE3‐positive findings corresponding to definitive atypical cells were identified. Although residual SCC with treatment‐related effects could not be excluded, marked degeneration made it difficult to determine whether the lesion was benign or malignant. (a, Hematoxylin and eosin staining, ×40; b, Hematoxylin and eosin staining, ×200; c, AE1/AE3 immunostaining, ×200). Figures (b) and (c) show higher‐magnification views of the boxed area in Figure (a). [file DEO2-6-e70328-s005.tif]

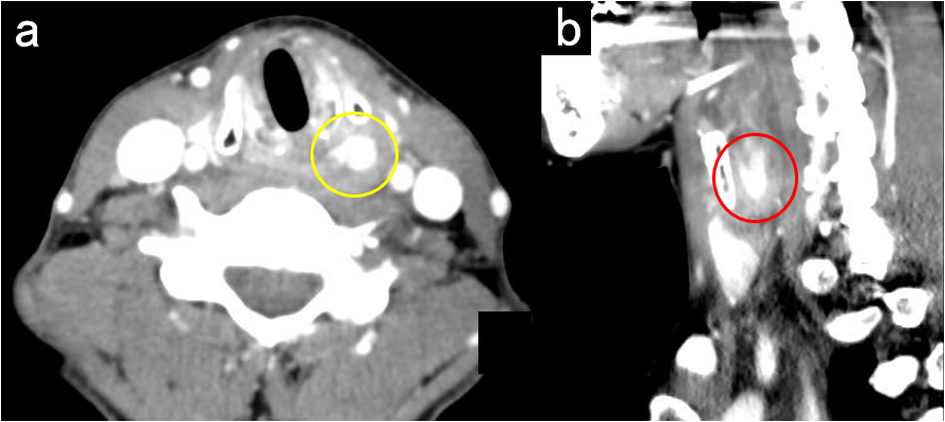

Supplement: Supplementary file 5 — Figure S5: Contrast‐enhanced computed tomography images. (c) Axial view showing an approximately 10‐mm enhancing mass (yellow circle). (d) Sagittal view showing an approximately 20‐mm enhancing mass (red circle). [file DEO2-6-e70328-s007.tif]

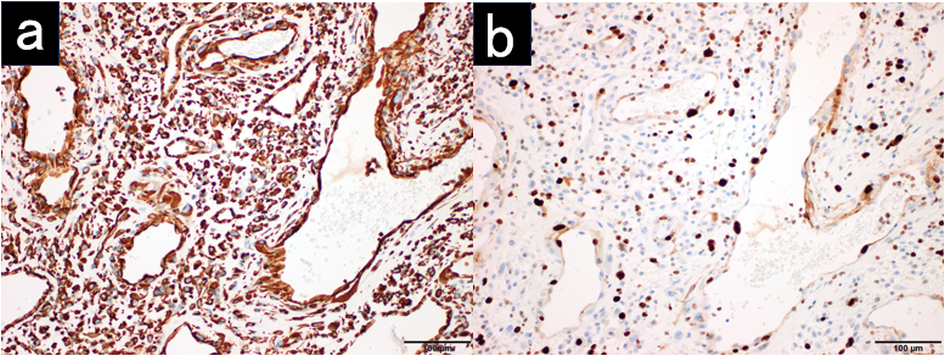

Supplement: Supplementary file 6 — Figure S6: Immunohistochemical findings of the stromal core of the inflammatory granulation tissue with atypical squamous epithelium and stromal cell proliferation. (a) Vimentin was diffusely positive (×200). (b) Numerous MIB1‐positive cells were observed (×200). [file DEO2-6-e70328-s001.tif]
